# Two Years' Experience of the Operation for Strangulated Hernia at the Bristol General Hospital: And Its Lessons

**Published:** 1896-03

**Authors:** Charles A. Morton

**Affiliations:** Surgeon to the Bristol General Hospital, and Demonstrator of Anatomy, University College, Bristol


### TWO YEARS' EXPERIENCE OF THE OPERATION FOR STRANGULATED HERNIA AT THE BRISTOL GENERAL HOSPITAL: AND ITS LESSONS.

BY

CHARLES A. MORTON, F.R.C.S. Eng.,

*Surgeon to the Bristol General Hospital, and Demonstrator of Anatomy, University College, Bristol.*

---

THE statistics of operations for strangulated hernia at St. Bartholomew's, St. Thomas's, and Guy's Hospitals up to 1884[1] show a mortality of 43 per cent.—a death-rate which, as Mr. Bowlby says, is greatly higher than is generally supposed. During the last ten years Mr. Bowlby has found that the mortality at St. Bartholomew's has been 35.8 per cent. He[2] attributes the still high mortality of the antiseptic period to delay. He thinks patients die from exhaustion, from the compulsory starvation of several days' duration, and the vomiting and pain, and hardly any from the operation itself. After three or four days' strangulation he considers the chances of recovery are but small. Some very valuable statistics bearing on this point have been worked out by Macready[3] from 129 of Reichel and Hagedorn's cases, all operated on aseptically:

| | | | | |
|---|---|---|---|---|
| 12.5 | per cent. | died after | one day's strangulation | or less. |
| 26.1 | ,, | ,, | two days' | ,, |
| 36.3 | ,, | ,, | three days' | ,, |
| 44 | ,, | ,, | four and five days' | ,, |

[1] *Lancet*, 1893, i. 1221. [2] *Loc. cit.* [3] *A Treatise on Ruptures*, 1893, p. 365.

He says: "The facts concerning the danger of delay have been long recognized by hospital surgeons, but that they have not yet reached all branches of the profession is evident from the heavy mortality still attending the operation. The patient who dies after twenty-four hours of strangulation, in most cases owes his fate to the negligence of his surgeon, and the latter, when he ventures to postpone the operation, is accepting a grievous responsibility."

During my first two years' work as surgeon to the Bristol General Hospital, I operated on twelve cases: two of these died, because they were sent in too late; all the others recovered. One of the fatal cases was so collapsed, I dared not give an anæsthetic, and operated under cocaine; the other was an aged woman who was sent in after a week's strangulation, and had not the vitality to recover.

Most surgeons would try taxis before operation, unless they had reason to fear gangrene of the bowel, and I have always done so, but never for long periods of time, believing prolonged taxis to be distinctly a dangerous proceeding, as likely to seriously bruise the distended bowel—even produce rupture of one or more of its coats—or bring about perforation of one of those small ulcers which are apt to form at the line of constriction. I generally try taxis for two or three minutes only before anæsthesia, and again for two or three minutes under anæsthesia, before operating; and often an ice-bag has been applied to the hernia during the interval between the patient's admission and my arrival at the hospital. In late cases where I had reason to suspect a serious condition of the bowel, I did not employ taxis. The ice-bag has not been successful in most of the cases under my own care; but I can record one very striking illustration of its value. A young man was admitted with a fairly large and recently strangulated inguinal hernia. I saw him before he went up to the ward, and tried taxis, but failed. When I saw him again, after having had an ice-bag on the rupture for one hour, the bowel had spontaneously slipped back. It was a very early case, the onset of symptoms being only five hours before admission.

I am, however, inclined to agree with those who think that if an ice-bag is used at all it should only be employed quite early in the case, within the first few hours; for, looking at the fact that gangrene begins very early in some cases, it seems to me a distinctly dangerous proceeding to apply intense cold to a coil of bowel the nutrition of which may be doubtful.

In several of my cases the bowel was tightly constricted by the ring, but only partially, *i.e.* only the uppermost coil in the ring, which was in contact with the sharp edge, was nipped, and the coil, passing through underneath, escaped, the constricting band being the uppermost portion of the ring only. In most cases of strangulated hernia the furrow on the bowel is most marked at one part, where the sharp edge of the ring has nipped it; but in these cases the undermost portion of bowel could hardly be said to be strangulated at all. In case No. 12 the finger could be passed into the abdomen through the ring (before its division), though the coil immediately beneath the sharp upper margin was deeply grooved by it, and I could hardly get my nail between the sharp margin of the saphenous opening and the bowel to act as a director for the introduction of the hernia knife. This condition of the hernia accounted for the fact that during taxis part of the hernia got very soft and part hard, for doubtless the fluid in the sac went back into the abdomen under pressure and left it softer, with thickened œdematous coils of bowel palpable. It is not, of course, meant that the ring was not completely occupied by the gut in this case, but that the finger easily passed between the bowel and the lower boundary of the ring by displacing the gut. In another case (No. 5), although the small bowel was much congested and grooved by the sharp upper margin of the external ring, against which it lay, yet beneath it the colon and a large mass of omentum passed out, and showed no signs of strangulation, and my finger could be passed into the abdomen, before division of the ring, underneath the bowel and omentum.

The remarkable pallor of the bowel associated with such marked œdema in the case of the old man of eighty-five (case 12) is very interesting. The constriction was very great, the sulcus being well marked where it had been nipped. It is an

### CASES OF STRANGULATED HERNIA.

| Name, Age, and Date. | Form of Hernia. | Duration of Strangulation. | Symptoms, &c. | Amount of Taxis. | Condition Found on Operation. | Condition after Operation. |
|---|---|---|---|---|---|---|
| 1. M. K. 63. Female June, 1893. | Fem. | 48 hours | Hernia for 4 years. Did not go back at night. Never wore truss. No increase in size lately. Vomiting for 48 hours. Said to have been fæcal for last 24 hours. No passage of fæces or flatus for 48 hours. Much pain in abdomen on second day. Local signs of strangulation except tension. | For doubtful time before admission and for a few minutes after. | Chocolate-coloured fluid in sac, and plum-coloured bowel, and omentum. Two ounces latter removed. Constriction at deep crural arch. Banks's radical cure. | Vomited before coming round from anæsthetic, and this was fæcal. Flatus passed in twelve hours. Bowels open on seventh day. Wound healed on eleventh. Discharged six days later. |
| 2. S. A. B. 58. Female. Dec., 1893. | Fem. | 16 hours | Ruptured many years. Never wore truss. Came down 46 hours before admission and could not be returned. Vomited three times. Abdominal pain. No flatus passed. All local signs of strangulation. | Ice-bag for three-quarters of an hour. Taxis for a few minutes before and again under anæsthetic. | Sac looked very black, and on opening it dark blood came out. Bowel very congested. Constricting band probably Hey's ligament. One-and-a-half ounces omentum removed. Banks's radical cure. | Diarrhœa came on on third day, and some suppuration in the wound was noticed. Diarrhœa ceased, and abdominal distension set in, and by the seventeenth day fæcal discharge from the wound was observed. This almost ceased, but distension of the abdomen became marked, though the bowels were open regularly and there was no sickness. The distention continued, and five months after operation an attack of complete obstruction set in, with visibly distended coils. This was relieved by passing a tube into the fæcal fistula After this, with very careful dieting, she remained very comfortable, hardly any disharge took place through the fæcal fistula and the abdominal distension diminished, and she was able to go about.* |

* I saw this patient in September of last year. She was looking so well I hardly recognised her. The fistula had quite closed and there was no abdominal distention, but she had occasional attacks of abdominal pain and vomiting lasting some hours.

| Name, Age, and Date. | Form of Hernia. | Duration of Strangulation. | Symptoms, &c. | Amount of Taxis. | Condition Found on Operation. | Condition after Operation. |
|---|---|---|---|---|---|---|
| 3. E. N. 41. Female. Jan., 1894. | Fem. | 24 hours | Ruptured 20 years. Never quite disappeared. Never wore truss. Severe abdominal pain and frequent vomiting. No flatus passed. | Ice-bag for four hours. A few minutes' taxis before and under anæsthetic. | Small knuckle of very congested gut, buried in omentum, seven ounces of which were removed. Constriction at crural ring. Banks's radical cure. | Bowels open the eighth day. Wound healed on eleventh. Discharged on fourteenth. |
| 4. Middle-age. Male. April, 1894. | Ing. | 7 hours | Ruptured 20 years. Always reducible. Never wore truss. During violent exertion, three hours before admission, came down much larger than usual. Great pain in abdomen. Vomited twice after admission. Hernia as large as a child's head. Local signs of strangulation. | Taxis for half-an-hour before I saw him. Ice-bag for four hours. Then taxis for several minutes, and again under anæsthetic. | Hernia was all bowel, which was considerably congested. Stricture at external ring. Ball's radical cure. | Bowels open a few hours after operation, and much red blood passed; and again four days after, when much black blood was passed. Flatulent distension of abdomen for many days after operation, relieved by rectal tube. Remained in hospital a month. |
| 5. Male. 64. June, 1894. | Ing. | 3 days | Three days before admission slipped, and rupture came down under the truss, and pain came on in abdomen, with vomiting. Bowels not open or flatus passed since. Latterly vomit said to have been fæcal. Hernia size of child's head. No tension, but no impulse. | Probably taxis tried some time before admission. I tried it for a few minutes under anæsthetic. | Hernia contained much small intestine and a portion of transverse colon, with a great quantity of omentum attached. Adhesions between coils were œdematous and hemorrhagic, and several patches of hemorrhage were seen under the peritoneum on the coils. The peritoneum tore off in a few places on even gentle manipulation where there had been hemorrhage beneath. Constriction at external ring. Half pound of omentum removed. | Passed flatus in twenty-four hours. No symptoms. |
| 6. E. T. 49. Female. | Fem. | 48 hours | Ruptured 5 years. Never wore truss. During violent exertion, 48 hours before admission, unusual amount descended, with pain in abdomen, which continued, and she vomited frequently. Hernia felt hard with exception of small area on surface of it, which was soft and fluctuating. No impulse. | Taxis for one hour before admission. | Thin-walled cyst in sac wall first opened. It contained fluid of different colour from fluid in the sac, but resembled sac before opening. Bowel dark, with deep sulcus. Banks's radical cure. | No symptoms. Discharged twenty-first day. |

| Name, Age, and Date. | Form of Hernia. | Duration of Strangulation. | Symptoms, &c. | Amount of Taxis. | Condition Found on Operation. | Condition after Operation. |
|---|---|---|---|---|---|---|
| **7.** Male. 76. Oct., 1894. | Ing. | 36 hours | Ruptured 50 years. Never wore truss. Unusual amount came down during defecation 36 hours before admission, and then he vomited. Bowels would not act. Was not sick after taking tea and bread and butter, followed by two hours' ride to Hospital, but vomited once after admission. Large swelling occupying left side of scrotum, and extending over external ring. Lower part felt like a hydrocele, except that it lay above the testicle and not around it. The upper, which was separated from the lower by constriction, lay over ring, and felt like a rupture. No impulse in either. | Taxis for fifteen minutes before I saw him, and for a few minutes under ether. | Lower part of the swelling contained several ounces of serous fluid; it was part of the sac into which the strangulated intestine could not descend. The upper swelling contained the strangulated bowel. Constriction at external ring. | No symptoms. Discharged fourteenth day. |
| **8.** F. T. 18 months. Male. Oct., 1894. | Ing. | 24 hours | Ruptured 6 months. Came down during defecation. Vomiting, and no action of bowels. Abdomen distended. Hernia size of large duck-egg. Local signs of strangulation. | Ice-bag for an hour. A few minutes' taxis before and under anæsthetic. Dresser also tried taxis for a few minutes. | Lower half of sac full of blood-stained serum only. A little blood-clot on bowel, and much in mesenteric attachment. Constriction at external ring. Not congenital sac. Banks's radical cure. | No symptoms. Discharged fourteenth day. |
| **9.** Female. 81. Dec., 1894. | Fem. | 1 week | Ruptured 40 years. Has come down under truss for several years. Omitted to support during defecation, and pain in abdomen came on directly after, and then vomiting. Vomiting lasted two days, and then ceased for four days, though she drank tea often. Vomited again on seventh day during removal to Hospital, and once after admission. No action of bowels or passage of flatus all the week, though she had aperients and enemata. Much distension of abdomen. Visible coils. Hernia soft, but no impulse. Pulse 120. Seemed very feeble. | Taxis before admission for uncertain period. After admission, a few minutes under anæsthetic. | Bowel only moderately congested. Well marked sulcus. Banks's radical cure. Operation well borne. | During recovery from anæsthetic vomited fæcal material, but not sick after. Flatus passed on third day; but bowels not open though enema given, as distension of abdomen continued, although there was no vomiting. She became more prostrate on night of third day, and died on morning of fourth. P.M.E.—Strangulated coil and neighbouring coil had very thin layer of lymph on them. In mucous membrane on inner aspect of one sulcus was a small ulcer. No sign of suppuration in herniotomy wound. Bowel above strangulated portion much distended. |

| Name, Age, and Date. | Form of Hernia. | Duration of Strangulation. | Symptoms, &c. | Amount of Taxis. | Condition Found on Operation. | Condition after Operation. |
|---|---|---|---|---|---|---|
| **10.** H. C. 54. Female. Dec., 1894. | Fem. | 24 hours | Ruptured for years. Twenty - four hours before admission came down when lifting heavy weight, and would not go back. Great pain in abdomen and vomiting, and no passage of fæces. Hernia consisted of two parts, one very soft and fluctuating, the other hard. No impulse. | Taxis a few minutes before and again under anæsthetic. | Two sacs: one projecting into the other. The soft fluctuating one contained only fluid; the other only bowel, which was strangulated. There were a few hemorrhages on it. Banks's radical cure. | Bowels open slightly same evening. No symptoms. Discharged in a month. |
| **11.** Female. 71. Dec., 1894. | Fem. | 3 days | Vomiting came on when laid up in bed with bronchitis. No action of bowels or passage of flatus. Did not notice hernia until second day of symptoms, and only called doctor's attention to it on third day. Never noticed it before. Admitted on third day in a too collapsed condition to give anæsthetic. Operation under cocaine. Abdomen distended. Local signs of strangulated hernia. Vomiting frequent and fæcal. | Taxis tried by doctor at home. Not again after admission. | Blood-stained offensive fluid in sac, looking like blood that had been mixed with strong carbolic lotion. Gut very dark and offensive, with a black patch on it, and two or three patches where the wall looked much thinned. No loss of lustre. One spot in sulcus of constriction looked very thin, as if an ulcer on the inside had nearly perforated. Gut left in sac, after division of ring. Operation only a quarter of an hour. | No further vomiting, but prostration continued, and she died four hours after operation. P.M.E.—Gut lay just as it had been left at operation. Coil above fairly distended. No perforation visible, but water slowly leaked from the bowel without any great pressure. Very shallow ulcer on inner aspect of deep sulcus, but not nearly perforating. The thinned patches were due to stretching apparently: there seemed little more than the peritoneal coat, but no actual perforation. The appearance on the outside resembled the protrusion of Descemet's membrane in a corneal ulcer, only it was the outer coat which protruded, not the mucous membrane of the bowel. There was no peritonitis. |
| **12.** A. H. 85. Male. June, 1895. | Fem. | 48 hours | Ruptured 10 years. Vomited twice in first 24 hours. Passed flatus during second 24 hours. Abdomen only moderately distended, but some distended coils seen. Irreducible femoral hernia on right side without impulse, and irreducible inguinal on left with impulse. No tension in either. Under taxis, part of the femoral hernia got softer and part harder. Operation on the femoral hernia. | Taxis for uncertain duration before admission. After admission, one and a half hours' ice-bag and then taxis for seven minutes, and then ice-bag again for 4 hours and a few minutes' taxis under anæsthetic. | Strangulated coils, pale colour but very œdematous. Serous fluid in sac. Constriction at saphenous opening. A little hemorrhage at mesenteric attachment. Banks's radical cure. | No shock from operation. On following day bowels open and a large amount of flatus passed. Discharged twenty-fifth day. |

interesting question why such marked œdema was associated with pallor rather than congestion. Possibly the great age of the patient, and his feeble circulation, may account for it.

The intermission of the vomiting in case 9 is exceptional. For four days the patient drank "sweet tea" and did not vomit, although she was not under the influence of opium. The same thing occurred, though to a less degree, in cases 7 and 12, in which, after the initial vomiting, the patients were able to keep down solid food. In all these cases the strangulation was marked. In cases of strangulated hernia, patients frequently only vomit if they take solid food or drink; but in these cases they were taking liquids or solids, and yet did not vomit. In case 1 the vomiting was fæcal by the end of the first forty-eight hours of strangulation, though the bowel was only congested and recovery was rapid.

In one case (No. 12) the patient had two irreducible herniæ, and the question arose as to which was strangulated. One was a femoral, the other an inguinal; both had an impulse on coughing, and neither was tense. But on dragging the femoral hernia downwards, away from the lower abdominal wall on which it lay, just above Poupart's ligament, the impulse ceased—it was only transmitted through the abdominal wall,—and this sign was found trustworthy, as the femoral hernia was operated on and found to be strangulated. The impulse in the strangulated hernia as it lay against the abdominal wall was, of course, not distensile.

In three of the cases I noticed the absence of tension in the hernia, which is one of the usual signs of strangulation, and due to the effusion of fluid into the sac or into the bowel. Macready[1] suggests that the rare condition in which the tension in strangulated hernia is absent may be due to such tight gripping of the bowel that both the arterial as well as the venous circulation in its walls would be at once suspended, and no fluid would be poured out either into the bowel or the sac. In case 5 the hernia was an inguinal one of the size of a child's head, and there was very little fluid. It contained small intestine, transverse colon, and a large mass of omentum. Almost

[1] *Op. cit.*, p. 344.

certainly fluid in the sac could have been pressed into the abdomen during taxis, as, although the bowel was tightly nipped where it passed under the upper margin of the ring, the finger could be passed into the abdomen underneath the bowel as already described. In case 12, where there was also no tension, there is evidence that the fluid did pass from the sac into the abdomen during taxis, though a fair amount remained in the hernia. In another, there were only a few drops of chocolate-coloured fluid in the sac. The bowel was plum-coloured, and recovered well. It was a small femoral rupture (case 1). But in case 9 there was a considerable quantity of clear serous fluid. The hernia was a small femoral one and the bowel only moderately congested, and there was hardly any omentum in the sac.

It will be noticed that the two fatal cases were those in which the nature of the case had been overlooked, and the strangulation was advanced. In all the cases where the patients had come early they recovered, and to their early admission their recovery is mainly due. Case 11 points to the advisability of always examining for hernia in cases presenting signs of intestinal obstruction. Had such an examination been made the hernia would have been discovered early, and the woman's life probably saved. To examine for hernia in intestinal obstruction is, of course, an established rule of surgery, but it may be well to refer to it in connection with this illustrative case.

The perfectly satisfactory use of cocaine for anæsthesia in cases where chloroform or ether cannot be given is well shown by the same case. I injected half a grain of cocaine in solution, along a line where the incision was afterwards made, in five places. The patient hardly felt the incision, and certainly nothing more of the operation, though she was quite sensible at the time. I put in three fishing-gut sutures, ready for tying as soon as the skin-incision had been made.

The resemblance of the hernia in case 7 to a combination of hernia and hydrocele was interesting. Over the region of the external ring was a firm dull swelling, the size of a rather flattened orange. This occupied quite the upper half of the left side of the scrotum. The lower part of the swelling felt like a soft hydrocele, and was separated from the upper part by a slight constriction; but the testicle lay beneath, and was not surrounded by it. The reason why the bowel only occupied the upper part of the sac, allowing the fluid to collect in such large quantities below, was that the strangulated coils were bound together by old adhesions, and could not probably descend as far as they had done at one time. The hour-glass constriction of the sac was not sufficient to prevent the descent of the bowel into the lower half.

In the case of the child (No. 8), the lower half of the sac contained fluid only, the upper held the strangulated bowel; but there was no constriction between the two, as in case 7, suggesting the combination of hydrocele and hernia. A somewhat puzzling condition found in case 6, was the cyst in the sac-wall. I thought when I opened it I was in the sac, and yet it was so small and no bowel could be found. It was not a pouch in the sac, as the fluid it contained was different in character from that in the sac, and no communication could be made out. These cysts are every now and then met with in operations on hernia and their nature is uncertain. Case 3 is an example of the division of the sac into two distinct parts by a band of fascia. The hernia was a femoral one: one part lay over the saphenous opening, the other over Poupart's ligament. Each was the size of an orange, and they moved together. The sac when exposed was found to have a constricting band of the superficial fascia passing across it, dividing it into two compartments. Both were full of omentum; but under the omentum in the lower sac was a knuckle of gut. In case 12 (an inguinal rupture) the sac was also partially divided by a band of fascia; both compartments contained bowel. Case 10 resembles case 5 to a certain extent. The hernia, a femoral one, consisted of two parts: one on the outer side, very soft and fluctuating; one on the inner, hard and tense. I first cut into the soft swelling, and found a sac of serous fluid, but no bowel, only a round mass of what looked like another very thick sac; and on cutting this open, I found the strangulated bowel. This was an example of double sac. The supposed cause of these double sacs is that the original sac becomes obliterated at its neck when the rupture has been kept up for years by a truss, and then fluid collects in the old sac; then, in the course of time, another sac gets pushed down and invaginated into the old sac, which has become filled with serous fluid; so that on operating on such a case, we first cut into the old sac and find serous fluid only, and then on opening the deeper sac, which projects into it, we discover the bowel. This was exactly the condition in my case; but unfortunately for this theory of causation she had never worn a truss, and the hernia had been constantly descending, so that there had been no chance for a hernial sac to become obliterated at its neck. In a case of Mr. Hulke's—quoted by Macready,[1]—the hernial sac was only discovered after cutting through a series of three cysts which lay over it, each cyst bulging into the one superficial to it.

In several of the cases, it will be noticed, there was blood in the fluid in the sac (case 2), or extravasation on the bowel, and in one case (5) there was a troublesome tendency for the peritoneum to peel off the bowel over the hemorrhagic patches on very gentle manipulation of the bowel in the hernial sac. In one case blood was not found in the sac or on the bowel (case 4), but was passed *per anum* in considerable quantity after operation. In only one case was I the only person who tried taxis, so that I cannot say how much force had been used; but in the case in which I alone tried it, I am perfectly certain no undue force was employed, and it was only continued for two minutes without anæsthesia and three minutes under. The hemorrhages were very trifling ones on the surface of the deeply congested coil. I am inclined to think hemorrhages under the peritoneum may take place, if not from congestion alone, from the very slightest manipulation. All the cases in which there was extravasation of blood on the bowel made very satisfactory recoveries. Mr. W. H. Bennett considers that slight extravasation of blood under the peritoneum is the rule rather than the exception after taxis has been employed.[2] I have seen the omentum infiltrated with blood-clot in a case of strangulated hernia operated on by another surgeon, but never such sanguineous fluid as in case 2. My assistant at the operation, thinking

[1] *Op. cit.*, p. 384. [2] *Lectures on Hernia*, 1893, p. 83.

it was blood from some large vessel, as it streamed out on opening the sac, at once seized a pair of forceps to catch the divided artery.

The remarkable thinning of the gut in patches without actual ulceration, described in the record of case 11, is very remarkable. They might be called extreme local bulgings of the bowel-wall. I cannot find any description of such a condition occurring in strangulated hernia.

The case of stricture following herniotomy (case 2) is of much interest. The conditions which give rise to intestinal obstruction after herniotomy have been well described by Mr. Treves.[1] They are: (1) adhesions of the bowel to the abdominal wall at the ring, or adhesions between the strangulated coils; (2) narrowing from the cicatrix of ulceration in the strangulated bowel. As a rule the symptoms do not come on for weeks after the patient has recovered from the herniotomy, and often not for a considerably longer period. The cause of the formation of the fæcal fistula in this case is not clear. There was no sign of any perforating ulcer in the sulcus of constriction when the bowel was returned, and though very congested, there was no question of gangrene either general or local in the coil. Although the wound suppurated from the first, no fæcal discharge was noticed until the seventeenth day after the operation: but it must be remembered that Banks's radical cure was done after the strangulated bowel had been returned; and the neck of the sac surrounded by the ligature came away with the discharge from the wound at the time the fæcal discharge was first noticed, so that perforation setting up suppuration in the wound may have started soon after the operation, and fæces only have found a way out when the neck of the sac became detached by suppuration later on. If so, there might have been an ulcer in the interior of the bowel (where ulcers always start) at the seat of constriction, which perforated the peritoneal coat after reduction; or a small patch of necrosis and sloughing on the bowel-wall may have formed. In this case there was persistent diarrhœa after the operation. This generally indicates enteritis, unless due to purgatives given before operation, but then it is not continuous.

[1] *Lancet*, 1884, i. 1021.

I did not operate on one of these twelve cases without the most careful and thorough examination of the sulcus in the bowel where it had been nipped by the ring, and for this purpose I invariably drew down the bowel a little way before returning it; for unless this precaution is taken—and it is not taken, I know, by all surgeons—I believe we are always in danger of returning into the abdomen bowel in the sulcus on which an ulcer may be just perforating, or have just perforated, for the perforation may be merely the size of a pin-hole and difficult to detect. Macready refers[1] to the case of a young man who within the last few years was operated on for strangulated hernia at St. Thomas's Hospital, only five hours after the onset of strangulation; but after division of the stricture, reduction was attempted without inspection of the line of constriction, when fæcal matter was seen coming from the neck of the sac; and when the bowel was drawn down, an opening was seen in it involving half its circumference.

It is a very interesting question why these ulcers at the line of constriction begin in the mucous membrane. It seems almost incredible that they should; for it is the peritoneum which bears the stress of the nipping. That they do start within—in the mucous membrane—the *post-mortem* examination of my two fatal cases distinctly demonstrates; and such is the general teaching on the subject. It has been suggested that the peritoneum is the most resisting structure, and the mucous membrane the least, and hence that the former resists the pressure the longest—the latter gives way first; and again, that although the mucous membrane is such a vascular structure, yet for that very reason, if its blood-supply is interfered with, it is more easily destroyed, as it is so dependent for its nutrition on a good supply of blood, and hence it ulcerates first. This reason is probably not the correct one, as it is hardly a question of cutting off the blood-supply to a minute definite area of gut-wall.

The age of the patient in case 8—eighteen months—is worthy of note. The rarity of strangulation in children, and the facility of reduction when it does occur, have long been recognised.

[1] *Op. cit.*, p. 374.

Mr. Marsh, in 1874, recorded[1] the first operation for strangulated hernia at the Great Ormond Street Hospital in twenty-three years.

In conclusion, I would like to say how much I am indebted for assistance in writing this paper to the information collected by Mr. Macready in his recent work on Hernia. I have said nothing about the radical cure of hernia—performed in most of these cases, and in several others which were not strangulated ; time and space do not allow.

